# Factors Associated with More Severe Disease in Infants and Children with Pertussis in the Post-Pandemic Era: A Single-Center Retrospective Study

**DOI:** 10.3390/pathogens14100969

**Published:** 2025-09-25

**Authors:** Antonio Gatto, Danilo Buonsenso, Eleonora Rulli, Mariya Prokopchuk, Giuseppe Zampino, Maurizio Sanguinetti, Michela Sali, Marilena La Sorda

**Affiliations:** 1 Department of Woman and Child Health and Public Health, Fondazione Policlinico Universitario Agostino Gemelli IRCCS, 00168 Rome, Italy; eleonora.rulli@gmail.com (E.R.); prokopchukmariya98@gmail.com (M.P.); giuseppe.zampino@unicatt.it (G.Z.); 2 Area Pediatrica, Dipartimento di Scienze della Vita e di Sanità Pubblica, Università Cattolica del Sacro Cuore, 00168 Rome, Italy; 3 Centro di Salute Globale, Università Cattolica del Sacro Cuore, 00168 Rome, Italy; 4 Dipartimento di Scienze Biotecnologiche di Base, Cliniche Intensivologiche e Perioperatorie-Sezione di Microbiologia, Università Cattolica del Sacro Cuore, 00168 Rome, Italy; maurizio.sanguinetti@unicatt.it (M.S.); michela.sali@unicatt.it (M.S.); 5 Dipartimento Scienze di Laboratorio ed Ematologiche, Fondazione Policlinico Universitario Agostino Gemelli IRCCS, 00168 Rome, Italy; marilena.lasorda@policlinicogemelli.it

**Keywords:** pertussis, *Bordetella pertussis*, children, COVID-19, post COVID-19

## Abstract

Pertussis remains a potentially severe respiratory illness in pediatric populations, especially in vulnerable groups. A severe outbreak has been reported in the post pandemic era, but it is uncertain if risk factors for severe disease may have changed compared with pre-pandemic years. We conducted a retrospective analysis of children diagnosed with *Bordetella pertussis* infection at a single tertiary pediatric center, from January 2023 to May 2024 Clinical and demographic variables were compared between outpatients and hospitalized patients to identify factors associated with disease severity. A total of 71 patients were included. Younger age (*p* < 0.001), lower body weight, (*p* = 0.0005) and lack of vaccination (*p* < 0.00001) were significantly associated with hospitalization. No significant differences were found regarding clinical symptoms, oxygen saturation, fever, or viral co-infection. Conclusion: Unvaccinated status, younger age, and lower weight appear to be key risk factors for hospitalization in pediatric pertussis, underlining the protective role of vaccination and the vulnerability of younger infants. These findings support targeted vaccination efforts and early risk stratification to prevent severe disease in vulnerable infants.

## 1. Introduction

Pertussis, or whooping cough, is a highly contagious respiratory infection caused by *Bordetella pertussis*, primarily affecting children. Despite long-standing global immunization programs, pertussis continues to cause significant morbidity, especially in infants too young to be fully vaccinated [1]. Over the past two decades, many countries have reported cyclical resurgences of pertussis, even in populations with relatively high vaccine coverage [2]. These trends may reflect a combination of waning immunity, suboptimal vaccine uptake among adolescents and adults, and the circulation of asymptomatic carriers [3].

The COVID-19 pandemic introduced additional complexity to the epidemiology of respiratory infections. Non-pharmaceutical interventions (NPIs) such as lockdowns, masking, and school closures led to a temporary but near-complete disappearance of pertussis circulation [4]. As NPIs were lifted, a resurgence of pertussis was observed across several countries, including Italy. This phenomenon, often referred to as “immunity debt,” may have resulted in a larger susceptible population due to the absence of natural exposure during the pandemic years [5].

In Italy, there is a theorical pertussis vaccination mandate for children since the age of 61 days with three vaccinations in the first year and catch-up doses at age 5 and every ten years. In the period 2010–2022, the highest incidence of pertussis in Italy was between 2016 and 2018, with 1.6 cases per 100,000 population. Conversely, 2021 was the year with the lowest figure, with an incidence of zero percent. In 2022, the incidence rate of pertussis in Italy was 0.1 cases per 100,000 population [6], although these figures may be strongly underreported compared to recent serological surveillance studies [7]. Recent reports from the post-pandemic period have highlighted a significant uptick in pertussis cases, with several severe outcomes including death in young infants, without addressing risk factors for more severe disease in the post-pandemic phase [8]. This pattern underscores the urgency of better understanding risk factors associated with severe pertussis, particularly hospitalization, to improve clinical management and public health planning. While prior studies have identified key predictors such as young age and unvaccinated status [9], regional and temporal variations necessitate updated, localized data. This is particularly true as the pandemic has disrupted several other infections, therefore it is uncertain if risk factors for severe disease may have changed compared with pre-pandemic years.

This study aims to identify clinical and demographic variables associated with greater disease severity in children diagnosed with pertussis, as measured by the need for hospitalization, in a single tertiary pediatric hospital in Rome, Italy.

## 2. Materials and Methods

We conducted a retrospective observational study at a tertiary university hospital in Rome, Italy, including all laboratory-confirmed Bordetella infections from children aged 0 to 18 years between January 2023 and May 2024. Diagnosis was primarily based on PCR testing performed on nasal swab at first evaluation of the children in the pediatric emergency department. Coinfections were patients having, in addition to a positive PCR for *B. pertussis* (Bordetella ELITe MGB Kit), also a positive molecular test for respiratory viruses on nasal swab.

Data collected included age, sex, weight, vaccination status, school attendance, clinical symptoms, oxygen saturation, and respiratory virus co-infections. Patients were classified as discharged from the Emergency Department (Discharged) or admitted in the pediatric ward (Admitted). Statistical analyses included Mann–Whitney U and chi-square tests with significance at *p* < 0.05. All contingency tables with zero counts were handled using the Haldane–Anscombe continuity correction (+0.5 to each cell) before computing odds ratios and confidence intervals. Sex distribution between groups was compared with Fisher’s exact test. All statistical analyses were performed using SPSS version 22.0 (IBM Corp., Armonk, NY, USA). A 95% confidence interval (CI) was used for all *p*-value calculations.

## 3. Results

The study included a total of 71 patients, 47.9% (n = 34) male and 52.1% (n = 37) female, aged between 20 days and 17.9 years, who presented with *Bordetella pertussis* disease (Table 1).

Comparing patients discharged after evaluation in the Pediatric Emergency Department (PS) to those hospitalized (PED), several significant differences emerged. Median age was lower in the PED group (56.0 months, range 25–132) compared to the PS group (120.5 months, range 28–123) (*p* < 0.001).

Body weight was also significantly lower in the PED group (median 4.5 kg, range 3.32–9.0) compared to the PS group (median 13.0 kg, range 4.05–57.0) (*p* = 0.0005). Regarding vaccination status, no vaccinated child required hospitalization. Using the Haldane–Anscombe correction, vaccination was strongly associated with reduced odds of admission (OR = 239.95, 95% CI 13.39–4301.15; Fisher’s exact *p* < 0.00001).

No statistically significant differences were found between the groups in terms of oxygen saturation at presentation (median SpO_2_ 98.0% in PED vs. 98.5% in PS; *p* = 0.801), fever presence (*p* = 0.168), cough (*p* = 0.699), or respiratory viral co-infection (*p* = 0.481).

Although school attendance was more common among outpatients (PS group), the difference did not reach statistical significance (*p* = 0.072).

## 4. Discussion

We performed this study to evaluate if the risk factors for more severe pertussis disease were changed in the post pandemic phase compared with pre-pandemic ages, as authors have speculated that SARS-CoV-2 may have impacted children’s immunity due to the infection or lockdowns [5] and, hypothetically, new risk factors could have emerged. Our findings reaffirm that younger age, lower body weight, and lack of vaccination are primary predictors of hospitalization in pediatric pertussis cases. Importantly, no vaccinated child in this cohort required hospitalization, highlighting the critical role of immunization in preventing severe outcomes. This aligns with prior studies demonstrating that while pertussis vaccines may not prevent infection entirely, they substantially reduce the risk of complications [10].

Clinical symptoms such as fever or cough did not predict hospitalization. This suggests that symptom severity is not a reliable early marker for risk assessment. Emergency and outpatient pediatricians should be aware of this limitation in clinical evaluation. These findings support existing literature indicating that host-related factors, particularly immune maturity and vaccination status, more accurately predict disease progression [11].

Despite concerns about post-pandemic co-infections, our data did not reveal significant differences in viral co-pathogens. This may reflect the waning impact of the immunity debt hypothesis on co-infection dynamics or limitations in viral panel testing.

Interestingly, although school attendance trended higher in non-hospitalized patients, it did not reach statistical significance. This may be due to higher vaccination rates in school-aged children or their generally more robust immune responses [12].

Pertussis outbreaks have also been reported in other Italian areas. In Tuscany, an average of 28 children and adolescents were hospitalised for pertussis every year between 2016 and 2019, a level that surged to 259 in 2024. More than half of these cases were among children ages 10 to 16, with babies making up just seven per cent of cases [13]. Other central Italian regions reported similar increases [14]. Italy is not the only country seeing a resurgence of whooping cough. Between January and March 2024, there were more than 32,000 cases reported across the European Union, Iceland, Liechtenstein, and Norway [15].

The protective association between vaccination and reduced severity of pertussis underscores the need for: need for (i) early infant immunization, including maternal Tdap vaccination during pregnancy to confer passive immunity to neonates [16], (ii) catch-up campaigns targeting under-vaccinated adolescents and young adults who may act as reservoirs [17], (iii) enhanced clinical awareness, especially during peak seasons or in post-pandemic rebounds, to identify at-risk infants early and reduce avoidable hospitalizations. Public health authorities must remain vigilant during inter-epidemic periods and ensure that vaccine coverage remains high, particularly in vulnerable populations [18]. Furthermore, the potential effects of pandemic-related immunity gaps should inform vaccination strategy and disease surveillance in the coming years.

Our study has limitations to be acknowledged. The study’s retrospective nature may introduce information bias due to reliance on electronic health records. The single-center setting limits generalizability; results may not reflect national or international patterns. The relatively small sample size, particularly in subgroup analyses (e.g., co-infections), may limit the statistical power to detect more nuanced associations. We don’t have information about the number of doses administered to patients. Lack of longitudinal follow-up data prevents assessment of longer-term outcomes post-discharge.

## 5. Conclusions

This study identifies, in line with the pre-pandemic era, unvaccinated status, younger age, and lower body weight as significant predictors of hospitalization among children with pertussis. In contrast, symptom presentation and viral co-infections were not associated with disease severity. These findings emphasize the importance of timely vaccination, including maternal vaccination and adult catch-up vaccination, to indirectly protect younger infants not eligible for vaccination (infants younger than 2 months) and may aid clinicians in triaging patients at higher risk for severe outcomes. These insights could inform both clinical practice and policy-making to prevent future pertussis-related morbidity in children.

## Figures and Tables

**Table 1:** Study population.

	Discharged	Admitted	*p*-Value
Sex (M = male, F = female)	M= 28 (19.88%), F = 20 (14.2%)	M = 6 (4.2%), F = 17 (12.07%)	0.009
Age (months)	118.6 ± 13.4 (median 120.5, range 28–123)	66.7 ± 40.7 (median 56.0, range 25–132)	<0.001
Weight (kg)	19.6 ± 17.6 (median 13.0, range 4.05–57.0)	5.3 ± 2.2 (median 4.5, range 3.32–9.0)	0.0005
SpO_2_ (%)	97.8 ± 2.5 (median 98.5, range 87.5–100.0)	97.7 ± 1.8 (median 98.0, range 95.0–100.0)	0.801
Vaccination *	48 (100%)	0	<0.00001
Fever	6 (12.5%)	1 (4.35%)	0.168
Cough	38 (79.2%)	21 (91.3%)	0.699
Respiratory virus	19 (39.6%)	10 (43.5%)	0.481
School attendance	38 (79.2%)	4 (17.4%)	0.072

* For the zero cell in vaccinated dmitted patients, the odds ratio was calculated using the Haldane–Anscombe correction (+0.5 added to all cells): OR = 239.95 (95% CI: 13.39–4301.15); statistical significance was assessed using Fisher’s exact test (*p* ≈ 4.1 × 10^−13^).

## Data Availability

Available upon request to the corresponding author.
